# Closed loop construction of hypoglycemia risk management for high risk neonates in mother infant rooming in settings: a retrospective study with an embedded clinical decision support system

**DOI:** 10.3389/fped.2026.1798686

**Published:** 2026-07-07

**Authors:** Limin Chen, Hui Shao, Beimeng Yu, Min Wang, Caixia Tian

**Affiliations:** 1 Department of Obstetrics, Shaoxing Maternity and Child Health Care Hospital (Maternity and Child Health Care Affiliated Hospital, Shaoxing University), Shaoxing, China; 2 Department of Infectology, Shaoxing Maternity and Child Health Care Hospital (Maternity and Child Health Care Affiliated Hospital, Shaoxing University), Shaoxing, China; 3 Department of Neonatology, Shaoxing Maternity and Child Health Care Hospital (Maternity and Child Health Care Affiliated Hospital, Shaoxing University), Shaoxing, China; 4 Department of Information Technology, Shaoxing Maternity and Child Health Care Hospital (Maternity and Child Health Care Affiliated Hospital, Shaoxing University), Shaoxing, China

**Keywords:** clinical decision support systems, clinical practice, hypoglycemia, mother infant rooming, neonate

## Abstract

**Objective:**

To evaluate the effectiveness of an intelligent clinical decision support system (CDSS) for neonatal hypoglycemia management in mother-infant rooming-in settings, and to dissect the differential hypoglycemia risk conferred by individual high-risk factors and their specific combinations under standardized surveillance.

**Methods:**

A multidisciplinary team developed a knowledge-driven CDSS grounded in national expert consensus, integrating automated maternal-neonatal risk identification, dynamic tiered monitoring reminders, and structured stratified management recommendations. Effectiveness was assessed using a pre-post self-controlled analysis (historical control: January–March 2024, *n* = 522; CDSS-implemented: April–June 2024, *n* = 417) and a concurrent parallel controlled analysis (non-CDSS wards: *n* = 389; CDSS wards: *n* = 352). Neonatal hypoglycemia was defined as blood glucose <2.2 mmol/L. Risk factor combination patterns were explored among 6,667 system-flagged high-risk neonates.

**Results:**

CDSS implementation significantly reduced hypoglycemia incidence in both the pre-post (5.76% vs. 11.88%, *P* < 0.05) and parallel (5.40% vs. 9.25%, *P* < 0.05) analyses. Under CDSS-managed surveillance, the overall hypoglycemia incidence in the high-risk cohort was 6.3%. Marked heterogeneity was observed: preterm birth (15.4%) and low birth weight (25.0%) carried the highest independent risks, while risk escalated non-linearly with specific factor combinations, reaching 18.8% in neonates with five concurrent factors. Serial monitoring demonstrated a sharp decline in hypoglycemia incidence from 6.1% at first measurement to ≤0.4% thereafter.

**Conclusion:**

The intelligent CDSS effectively reduces neonatal hypoglycemia in rooming-in settings. Hypoglycemia risk depends more on the specific types and combinations of high-risk factors than on their quantity alone, providing evidence for precise risk stratification. This closed-loop, guideline-driven workflow enhances clinical standardization and patient safety. Future multicenter studies incorporating machine learning and long-term neurodevelopmental follow-up are warranted.

## Introduction

Neonatal hypoglycemia, the most prevalent metabolic disorder in the perinatal period, remains a critical focus in neonatal medicine. Epidemiological data indicate that the incidence of hypoglycemia in high-risk neonates (e.g., infants of diabetic mothers, small-for-gestational-age infants) can reach 30%–40% (1). Failure to promptly identify and manage persistent hypoglycemia may lead to permanent neurological damage and adversely affect long-term cognitive development (2). Evidence-based international guidelines recommend standardized glucose monitoring and management protocols for high-risk newborns (3); however, the implementation of these recommendations in routine clinical practice is often suboptimal, largely due to variations in healthcare systems, staffing resources, and clinical workflows (4).

Under the conventional mother-infant rooming-in care model, the screening and management of neonatal hypoglycemia faces several challenges. Clinical practice heavily relies on the individual experience, memory accuracy, and adherence of healthcare providers. Nurses must manually identify at-risk neonates from the general newborn population, recall dynamic monitoring schedules, and make clinical decisions by applying complex, multi-branch management pathways based on point-of-care glucose values. In the busy, multitasking environment of the maternity ward, this process is prone to monitoring omissions, delayed interventions, or deviations from protocol, posing a significant patient safety risk (5). Furthermore, the management process involves multi-disciplinary collaboration among obstetrics, pediatrics, and nursing departments, where variations in practice patterns and communication efficiency directly impact the quality of care delivered.

In recent years, clinical decision support systems (CDSS) have emerged as a major focus in healthcare informatization, offering innovative solutions for optimizing standardized clinical pathways. Evidence suggests that a well-designed CDSS can integrate electronic health record data to deliver personalized, evidence-based decision support to clinicians at the point of care, effectively reducing medical errors and enhancing patient safety (6). Key characteristics of successful CDSS include automated, rather than manual, triggering; seamless integration into the clinical workflow; and precise support at critical decision points (7). From a quality improvement perspective, such systems translate paper-based guidelines into executable, monitorable clinical actions, promoting the standardization of healthcare delivery (8). However, the successful integration and sustained adoption of such technological innovations within complex healthcare settings represent an implementation science challenge, which requires overcoming technical, organizational, and workflow barriers (9). Nevertheless, existing research on the digital management of neonatal hypoglycemia reveals notable gaps. Most studies have focused on developing risk prediction models for use in the neonatal intensive care unit (NICU), while research specifically tailored to the mother-infant rooming-in context remains relatively scarce (10, 11) . Furthermore, many existing systems are limited to single-point reminders and fail to achieve a full-cycle, closed-loop management process from risk identification to stratified intervention (12). Most importantly, even with intelligent management in place, there is a paucity of high-quality evidence—derived from large-scale, standardized data—to elucidate the differential risk of hypoglycemia among neonates with various high-risk factors (e.g., infants of diabetic mothers alone vs. those also small for gestational age) under standardized monitoring protocols.

To address these gaps, our multidisciplinary team developed and deployed a knowledge-driven intelligent decision support system tailored to neonatal hypoglycemia management in mother-infant rooming-in settings. Grounded in national expert consensus, the system translates complex, multi-branch clinical pathways into an automated, closed-loop workflow comprising three integrated modules: real-time risk identification through automated extraction of maternal-neonatal data, dynamic monitoring with tiered reminders delivered at nursing workstations and mobile terminals, and structured decision support that generates stratified management recommendations based on transmitted blood glucose values. Over a 2-year period of stable operation, this system has enabled standardized glucose surveillance in over 6,600 high-risk neonates, yielding a large, high-quality clinical database. To rigorously evaluate the system's effectiveness, a comparative study incorporating both pre-post self-controlled and concurrent parallel control designs was conducted. Leveraging this unique resource, the present study pursues two objectives. First, we aim to characterize the overall incidence and clinical features of neonatal hypoglycemia under this protocol-driven, mandatory standardized management in a rooming-in setting. Second, we seek to dissect the differential hypoglycemia risk conferred by individual high-risk factors and their specific combinations, thereby providing empirical evidence to guide precise clinical risk stratification and judicious allocation of monitoring resources.

## Methods

### Composition of the multidisciplinary research team

The research team comprises members from nursing, clinical medicine, computer science, and other disciplines, and was formed to develop a knowledge-based high-risk neonatal hypoglycemia nursing decision support system. This decision support system employs a hybrid network architecture and is embedded in the mobile nursing documentation system. The intelligent decision support system was developed in collaboration with an external software company. The clinical content, knowledge rules, and workflow requirements were defined by our multidisciplinary research team, while the software architecture and coding were implemented by the company's engineers to meet the hospital's specific clinical needs. To support effective management of neonatal hypoglycemia, an integrated clinical data platform was first designed to consolidate multi-dimensional information, including real-time blood glucose data, monitoring device data, and medical history records. This study was approved by the Pediatric Research Ethics Board of Shaoxing Maternity and Child Health Care Hospital (approval number: IRB-AF-002-01.1).

### Clinical setting and routine neonatal care

The study was conducted in the mother-infant rooming-in wards of a tertiary maternity hospital. In routine clinical practice, all newborns admitted to the rooming-in wards receive an initial physical examination by a pediatrician within 24 h of birth and a re-examination prior to discharge. A neonatologist is not physically stationed in the maternity ward at all times; rather, a pediatrician or neonatologist is consulted when clinical concerns arise, such as persistent hypoglycemia, suspected infection, or the need for neonatal intensive care unit (NICU) transfer. Nursing staff are responsible for executing routine newborn care, performing point-of-care blood glucose monitoring as ordered or as prompted by protocols, and implementing initial feeding interventions.

### Development and functionality of the intelligent decision support system

Based on the “Expert consensus on standard clinical management of neonatal hypoglycemia in China (2021)” (13), the research team established the content for the knowledge base. This consensus, developed by the Group of Neonatology of the Pediatric Society of the Chinese Medical Association, represents the first nationally unified, evidence-based guideline for neonatal hypoglycemia management in China, and applies to neonates with a gestational age of ≥35 weeks. As an English-language version of this guideline is not currently available, we provide a detailed summary of the key recommendations that informed the CDSS knowledge base and decision rules. The consensus establishes a clinical treatment threshold of blood glucose <2.6 mmol/L (≈47 mg/dL) and recommends that asymptomatic high-risk neonates undergo the first blood glucose measurement within 2 h of birth (optimally 30 min after the first effective feeding), with subsequent routine monitoring before feedings. For initial blood glucose values between 2.0 and 2.6 mmol/L in rooming-in neonates, the guideline recommends supplemental feeding followed by a recheck at 30 min: if the post-feed value is <2.2 mmol/L, NICU admission with intravenous (IV) dextrose is indicated; if between 2.2 and <2.6 mmol/L, continued supplemental feeding is advised, with NICU admission recommended if glucose remains <2.6 mmol/L after two supplemental feeds. For initial values <2.0 mmol/L, or for symptomatic infants with glucose <2.6 mmol/L, immediate NICU admission with IV dextrose (10% dextrose, 2 mL/kg bolus at 1 mL/min, followed by a glucose infusion rate of 5–8 mg/kg/min) is recommended. Target blood glucose ranges are defined as 2.8–5.0 mmol/L within the first 48 h of life and 3.3–5.0 mmol/L after 48 h. Glucose monitoring may be discontinued after three consecutive pre-feed values exceeding these targets, with the monitoring duration stratified by the initial risk factors: approximately 12 h for neonates whose sole risk factor is gestational diabetes mellitus; 24 h for most other risk factors (including SGA, preterm birth, and low birth weight); and 48 h for LGA and macrosomia. The glucose thresholds adopted in the CDSS—namely, <2.2 mmol/L as the critical threshold for NICU transfer, 2.2–2.6 mmol/L as the intermediate range requiring supplemental feeding and reassessment, 2.6–3.3 mmol/L as the range prompting intensified (3-hourly) monitoring, and ≥3.3 mmol/L as the threshold for routine (6-hourly) surveillance—were directly derived from the Chinese national consensus and are consistent with the range of operational thresholds endorsed by major international guidelines. The value of 2.6 mmol/L as an operational threshold for clinical intervention has been adopted by numerous national guidelines and was used as the treatment target in the CHYLD (Children with Hypoglycaemia and Their Later Development) prospective cohort study, which demonstrated that among at-risk neonates systematically screened and treated to maintain glucose ≥2.6 mmol/L, exposure to neonatal hypoglycemia was not associated with lower academic achievement at 9–10 years of age (14). The 2.2 mmol/L threshold for urgent intervention, including NICU transfer, aligns with the American Academy of Pediatrics (AAP) recommendation for immediate intravenous glucose in symptomatic infants with glucose <2.2 mmol/L (<40 mg/dL), and the 3.3 mmol/L threshold for routine surveillance aligns with the Pediatric Endocrine Society (PES) recommendation that at-risk neonates maintain glucose >3.3 mmol/L (>60 mg/dL) after 48 h of life (15). It should be emphasized that these values are operational thresholds—action triggers designed to prompt intervention before potentially harmful low glucose concentrations cause neurological injury—rather than diagnostic definitions of hypoglycemia. As noted in a recent comprehensive review of international guidelines, no single absolute glucose value defines clinically significant hypoglycemia for all newborns, and operational thresholds vary across guidelines (commonly ranging from 2.0 to 2.6 mmol/L in the first 48 h), reflecting the absence of a universal consensus (15, 16). The selection of these thresholds for the CDSS was therefore grounded primarily in the Chinese national consensus, with corroborating support from the range of thresholds endorsed by other major pediatric and endocrine societies, ensuring that the system's decision logic reflects a conservative, safety-oriented synthesis of available international evidence. The team determined the management process for high-risk neonates with a gestational age of 35 weeks or more and designed a multi-dimensional intelligent management model, including screening for high-risk neonates, preventive measures, early identification of hypoglycemia symptoms, blood glucose monitoring reminders, automatic transmission of POCT data, warnings for abnormal blood glucose levels, and standardized management suggestions. The intelligent decision support system is seamlessly embedded within the clinical workflow to provide closed-loop risk management for neonatal hypoglycemia in mother-infant rooming-in settings. Its core functionality consists of three intelligent modules:

1. Risk Identification Module: The screening eligibility criteria for identifying high-risk neonates were based on the aforementioned expert consensus. Neonates were classified as high-risk for hypoglycemia if they had any of the following maternal, fetal, or neonatal indications. Maternal indications included: gestational diabetes mellitus, gestational hypertension, thyroid disorders during pregnancy, obesity in pregnancy (pre-pregnancy BMI ≥28 kg/m^2^ based on Chinese standards, corresponding to ≥30 kg/m^2^ for international reference), and pre-delivery use of specific medications within 24 h (beta-blockers, dexamethasone, sulfonylurea oral hypoglycemic agents, and antidepressants). Fetal indications comprised: intrauterine growth restriction (diagnosed prenatally as described below), intrapartum hypoxia (defined as a 5-minute Apgar score <7 or umbilical artery pH <7.1), large for gestational age (birth weight >90th percentile), small for gestational age (birth weight <10th percentile), and macrosomia (birth weight ≥4,000 g). Neonatal indications included: preterm birth (gestational age ≥35 and <37 weeks), low birth weight (<2,500 g), and the development of clinical signs suggestive of hypoglycemia within 48 h of birth (e.g., jitteriness, lethargy, hypothermia, poor feeding, or weight loss exceeding 8%). Neonates could fulfill criteria through multiple indications across these categories, and all applicable risk factors were recorded and flagged independently by the CDSS. The corresponding monitoring schedules (timing of first and subsequent glucose measurements, frequency of monitoring, and criteria for discontinuation of surveillance) are detailed in the “Glucose Monitoring Protocol” subsection; the standardized feeding management (including the BFHI-based protocol for breastfeeding, supplementation, and use of oral dextrose gel) is described in the “Data Collection and Statistical Analysis” section; and the escalation pathways (including the process by which CDSS recommendations for NICU transfer are reviewed and authorized by a physician) are specified in the “Implementation of CDSS-Generated Recommendations in Clinical Practice” subsection. The risk identification mechanism for high-risk factors is implemented through real-time extraction of clinical data from the neonatal birth record form, which the system directly retrieves and integrates at the time of birth. To ensure clarity and avoid ambiguity in risk factor classification, the following standardized definitions were adopted and encoded into the CDSS. Large for gestational age (LGA) was defined as birth weight >90th percentile for gestational age and sex based on Chinese neonatal birth weight reference curves. Macrosomia was defined as birth weight ≥4,000 g, regardless of gestational age. Small for gestational age (SGA) was defined as birth weight <10th percentile for gestational age and sex. Intrauterine growth restriction (IUGR), also referred to as fetal growth restriction (FGR), was defined as a pathological condition characterized by fetal growth below its expected potential, diagnosed prenatally by obstetric ultrasound (estimated fetal weight <10th percentile or abdominal circumference <5th percentile with abnormal umbilical artery Doppler findings) and confirmed at birth. These categories were not mutually exclusive; a neonate could simultaneously fulfill criteria for multiple classifications. For example, an infant with a birth weight of 4,100 g at 38 weeks' gestation could be classified as both LGA (>90th percentile) and macrosomic (≥4,000 g), while a 2,500 g infant at 40 weeks could simultaneously meet criteria for both SGA (<10th percentile) and IUGR (if prenatal evidence of growth restriction was documented). This non-exclusivity was deliberate, as each category reflects a distinct clinical construct—percentile-based anthropometric deviation, absolute weight excess, and pathological growth failure, respectively—and each may confer independent hypoglycemia risk through different physiological mechanisms. The CDSS was programmed to flag all applicable risk factors for each neonate based on these definitions, and the subsequent risk factor combination analyses accounted for the potential overlap among categories by treating each factor as a separate binary variable. Specifically, the system consolidates biometric parameters of the neonate (including gestational age, birth weight, and Apgar score) with clinical background information of the mother (comprising pre-existing medical conditions, pregnancy complications, and intrapartum medication history). Pre-delivery medications within 24 h were defined as maternal use of the following medications within 24 h before delivery, particularly during labor: beta-blockers, dexamethasone, sulfonylurea oral hypoglycemic agents, and antidepressants. Through intelligent matching and logical analysis of these paired maternal-neonatal datasets, the system automatically recognizes high-risk indicators from both maternal and neonatal data and labels the neonate as ‘high-risk for hypoglycemia', triggering a prominent pop-up alert on both the nursing station workstation and mobile nursing terminals (PDAs). The alert explicitly states “Please monitor blood glucose within 2 h after birth” and displays the specific high-risk factors alongside preventive feeding recommendations. Gestational diabetes mellitus (GDM) was diagnosed according to a 75 g oral glucose tolerance test (OGTT) at 24–28 weeks of gestation, with any one of the following plasma glucose thresholds met or exceeded: fasting ≥5.1 mmol/L, 1-hour ≥10.0 mmol/L, or 2-hour ≥8.5 mmol/L. The system also applies to neonates who were not initially identified as high-risk at birth but develop abnormal signs within 48 h after delivery. Should any clinical manifestation suggestive of a high-risk status occur in the neonate (e.g., suspected hypoglycemia symptoms, hypothermia, or weight loss exceeding 8%), nurses are required to immediately perform point-of-care blood glucose measurements. Upon entry of the exact time of this blood glucose measurement into the system, the system automatically triggers the reassessment protocol. Based on these data, the system reclassifies the neonate as “high-risk for hypoglycemia”, initiates a comprehensive re-evaluation process, and issues targeted alerts along with subsequent clinical decision support.
2. Dynamic Monitoring and Tiered Reminder Module: This module generates standardized pop-up alerts on both computer and PDA terminals 10 min before each scheduled monitoring time point as per individualized management plans. The reminder, “Scheduled monitoring time has arrived, please measure blood glucose promptly,” ensures nurses complete monitoring tasks without omission amidst dynamic clinical duties.
3. Structured Decision Support Module: As the system's core, this module activates automatically once the blood glucose value is transmitted to the system. It first integrates the value into an individualized glucose trend graph, and then provides stratified management advice based on preset, evidence-based rule logic. The detailed decision logic is provided in Supplementary File S1 (titled “Clinical Pathway Decision Rules”): a) For a blood glucose level (BGL) below 2.2 mmol/L, it prompts “Immediate transfer to the Neonatal Intensive Care Unit (NICU).” b) For a BGL between 2.2 and 2.6 mmol/L, the system interactively queries for clinical symptoms. If symptoms are present, it recommends transfer; if absent, it guides the user to “Provide supplemental feeding and recheck blood glucose 30 min post-feeding,” followed by a stepwise assessment of the post-feed value (transfer if still <2.2 mmol/L; re-feed and recheck if 2.2–2.6 mmol/L; transfer if BGL remains <2.6 mmol/L after two supplemental feeds). c) For a BGL between 2.6 and 3.3 mmol/L, it advises “Monitor pre-feed blood glucose in 3 h.” d) For a BGL ≥3.3 mmol/L, it recommends “Monitor pre-feed blood glucose in 6 h.” Finally, based on the initial risk factors, the system can automatically deactivate blood glucose monitoring reminders at 12 h for gestational diabetes mellitus; at 24 h for gestational hypertension, thyroid disorders in pregnancy, obesity in pregnancy, pre-delivery medications within 24 h, intrauterine growth restriction, intrapartum hypoxia, small for gestational age, preterm birth, and low birth weight; and at 48 h for large for gestational age and macrosomia.

The alerting function of the system is designed for all mother-infant shared wards across the hospital. Access permissions are strictly allocated based on ward units; for instance, nurses working in Ward 1 of the Obstetrics Department are unable to view alert notifications from Ward 2. Alerts are simultaneously displayed on all computers within the ward as well as on handheld Personal Digital Assistants (PDAs). Each on-duty nurse carries a dedicated PDA. Notifications are not limited to pop-up windows; they are also accompanied by audible alerts and vibration to ensure that the responsible nurses do not miss critical information. If a specific user dismisses an alert, it only deactivates the notification on that individual's computer and PDA, without affecting other users. According to the notification mechanism, if an issued alert is not addressed by a nurse within 10 min, it is automatically relocated to the top-right corner of the system interface for persistent display and marked as unread. Through the coordinated operation of these three modules, the system translates a complex clinical management pathway into a standardized, automated, and traceable intelligent workflow, effectively mitigating the risk of human error and ensuring the homogeneous execution of the management protocol.

### Glucose monitoring protocol

All blood glucose measurements were performed using point-of-care testing (POCT) devices on heel-stick capillary samples. The first blood glucose measurement was mandated within 2 h of birth, and was obtained prior to the first feed to establish a baseline glucose level before enteral intake. Subsequent measurements were also scheduled as pre-feed assessments. The monitoring frequency was dynamically determined by the CDSS based on the most recent blood glucose value according to the following tiered protocol: (1) if the pre-feed blood glucose was ≥3.3 mmol/L, the next measurement was scheduled in 6 h; (2) if the blood glucose was between 2.6 and 3.3 mmol/L, the next pre-feed measurement was scheduled in 3 h; and (3) if the blood glucose was between 2.2 and 2.6 mmol/L and the neonate was asymptomatic, supplemental feeding was provided and a repeat measurement was performed 30 min post-feed, with further action determined by the post-feed value. Glucose surveillance was automatically discontinued by the CDSS once the neonate achieved stable normoglycemia over a predetermined monitoring period based on the initial high-risk factors: at 12 h of age for neonates whose sole risk factor was gestational diabetes mellitus; at 24 h for those with gestational hypertension, thyroid disorders in pregnancy, obesity in pregnancy, pre-delivery medications within 24 h, intrauterine growth restriction, intrapartum hypoxia, small for gestational age, preterm birth, or low birth weight; and at 48 h for those with large for gestational age or macrosomia. Neonates who developed any clinical signs suggestive of hypoglycemia (e.g., jitteriness, lethargy, hypothermia, or poor feeding) at any point underwent immediate additional point-of-care glucose testing regardless of the scheduled monitoring plan.

### Implementation of CDSS-generated recommendations in clinical practice

When the CDSS generates a management recommendation, the bedside nurse receives the alert simultaneously on the ward computer and the handheld PDA. The recommended actions are differentiated based on urgency and clinical requirement. For feeding-related recommendations (e.g., “Provide supplemental feeding and recheck blood glucose in 30 min” or “Monitor pre-feed blood glucose in 3 h”), the nurse may directly proceed with the intervention according to established nursing protocols and feeding guidelines, without requiring additional physician authorization, and document the action in the system. For critical alerts that recommend NICU transfer (blood glucose <2.2 mmol/L or symptomatic hypoglycemia with blood glucose 2.2–2.6 mmol/L), the nurse immediately notifies the attending pediatrician or the on-call neonatologist. The physician then evaluates the neonate at the bedside, makes the final clinical decision regarding NICU admission, and issues the corresponding medical order. The nurse executes the transfer only after the physician's order is documented in the electronic medical record. Thus, the CDSS functions as a real-time clinical decision aid, ensuring that standardized, evidence-based prompts are delivered at the point of care, while all critical clinical decisions and NICU transfers remain under the direct supervision and authority of a physician.

### System verification and testing

The system was validated through a simulation study conducted in a tertiary hospital. Real-world data were collected from mother-infant rooming-in wards, and a three-month clinical pilot was carried out. To rigorously evaluate the effectiveness of the CDSS, the study employed two complementary comparative designs: 1) Pre-Post Self-Controlled Analysis: Data were extracted from two comparable mother-infant rooming-in wards during January to March 2024, prior to CDSS implementation, to serve as a historical baseline. These data were then compared with data from the same wards after CDSS implementation, from April to June 2024, constituting a pre-post self-controlled analysis. During the pre-implementation phase, routine manual decision-making and conventional neonatal hypoglycemia nursing management were performed. In the post-implementation phase, the CDSS was introduced. Through operational demonstrations and system user guides, nurses were thoroughly familiarized with the system workflow, operating procedures, and module functions. Based on nurse feedback during this pilot phase, adjustments were made to the system interface (e.g., language, color scheme, and layout). 2) Concurrent Parallel Control Analysis: To control for potential temporal confounders inherent in the pre-post design, two additional obstetrics wards, where CDSS implementation was delayed, were selected to serve as a concurrent control group. In these wards, routine manual decision-making continued without the CDSS, while two other wards with timely CDSS implementation formed the study group. This design allowed for a direct, simultaneous comparison between the CDSS and non-CDSS management strategies from April to June 2024. To support implementation and gather qualitative feedback, a WeChat group was established, bringing together all participating nurses and multidisciplinary team members to discuss issues encountered during system use and potential solutions, thereby improving system usability. Finally, the distribution of risk factors and the incidence of neonatal hypoglycemia were compared between the groups in both designs to determine whether the implementation of the CDSS was effective.

### Data collection and statistical analysis

This study utilized two overlapping cohorts. Cohort 1 (*n* = 1,680) comprised high-risk neonates from four mother-infant rooming-in wards (January–June 2024) for the two comparative effectiveness analyses (pre-post and parallel controlled). Cohort 2 (*n* = 6,667) comprised all high-risk neonates managed by the CDSS from January 2024 to December 2025 across all mother-infant rooming-in wards, which includes the neonates from the CDSS-implemented arms of Cohort 1 plus all additional high-risk neonates managed by the system after June 2024 through December 2025. Cohort 2 was used for the risk factor combination and serial glucose monitoring analyses. All neonates met the high-risk criteria of the national expert consensus, had a gestational age ≥35 weeks, and were admitted to mother-infant rooming-in wards. Baseline characteristics of Cohort 2 are summarized in Supplementary File S2. All study data were extracted from the backend database of the intelligent management system and the associated hospital electronic medical record system. The data dimensions collected included: all categories of hypoglycemia risk factors identified by the system; all point-of-care blood glucose measurements, measurement frequency, and corresponding time points during the mother-infant rooming-in period; and the stratified management recommendations automatically triggered by the system based on blood glucose values. Regarding missing data management, all core variables analyzed in this study (including maternal and neonatal baseline characteristics, twelve established risk factors for neonatal hypoglycemia, and blood glucose monitoring results) were derived from required fields in the electronic medical record, with a completeness rate approaching 100%; therefore, no imputation for missing values was performed. It is important to note that all enrolled high-risk neonates received standardized early care, including early skin-to-skin contact and early sucking/breastfeeding initiated as soon as possible after birth (no later than one hour postpartum). This standardized feeding initiation protocol served as the common foundation for blood glucose management in this study. All enrolled neonates were managed according to a standardized feeding protocol grounded in the Baby-Friendly Hospital Initiative (BFHI) principles—our maternity ward holds full Baby-Friendly certification and adheres to the “Ten Steps to Successful Breastfeeding.” Early initiation of breastfeeding was promoted for all healthy neonates, with the first feed encouraged within one hour of birth. Exclusive breastfeeding was the default feeding modality for all mother-infant dyads without contraindications. When maternal breast milk was insufficient or when neonates exhibited clinical signs of hypoglycemia, expressed maternal milk was provided as the first-line supplement, followed by donor human milk when available, or commercial infant formula if neither was accessible. For neonates with blood glucose levels between 2.2 and 2.6 mmol/L who were asymptomatic, the protocol-directed intervention consisted of supplemental feeding (breastfeeding, expressed maternal milk, or formula as dictated by maternal preference and milk availability) followed by a repeat blood glucose measurement 30 min post-feed. Oral dextrose gel was not routinely used in our institution during the study period and was not integrated into the CDSS decision logic. The same feeding principles extended to the NICU setting: upon transfer for hypoglycemia, neonates received intravenous dextrose if clinically indicated, with enteral feeding (expressed maternal milk, donor human milk, or formula) initiated or resumed as soon as tolerated. Because institutional BFHI policies may independently influence hypoglycemia outcomes, these feeding practices formed the consistent clinical substrate upon which the CDSS intervention was evaluated. Neonatal hypoglycemia was defined as any point-of-care blood glucose measurement below 2.2 mmol/L during the mother-infant rooming-in period (17). All statistical analyses were performed using R software (version 4.3.0). Continuous variables with a normal distribution were presented as mean ± standard deviation (Mean ± SD), and comparisons between two groups were conducted using the independent two-sample *t*-test. Categorical variables were presented as frequencies and percentages (*n*, %), and group comparisons were performed using the chi-square test, or Fisher's exact test when the expected frequency was less than 5. All hypothesis tests were two-sided, and a *P*-value <0.05 was considered statistically significant.

## Results

### Introduction to the decision support system in use

**Figure 1:**
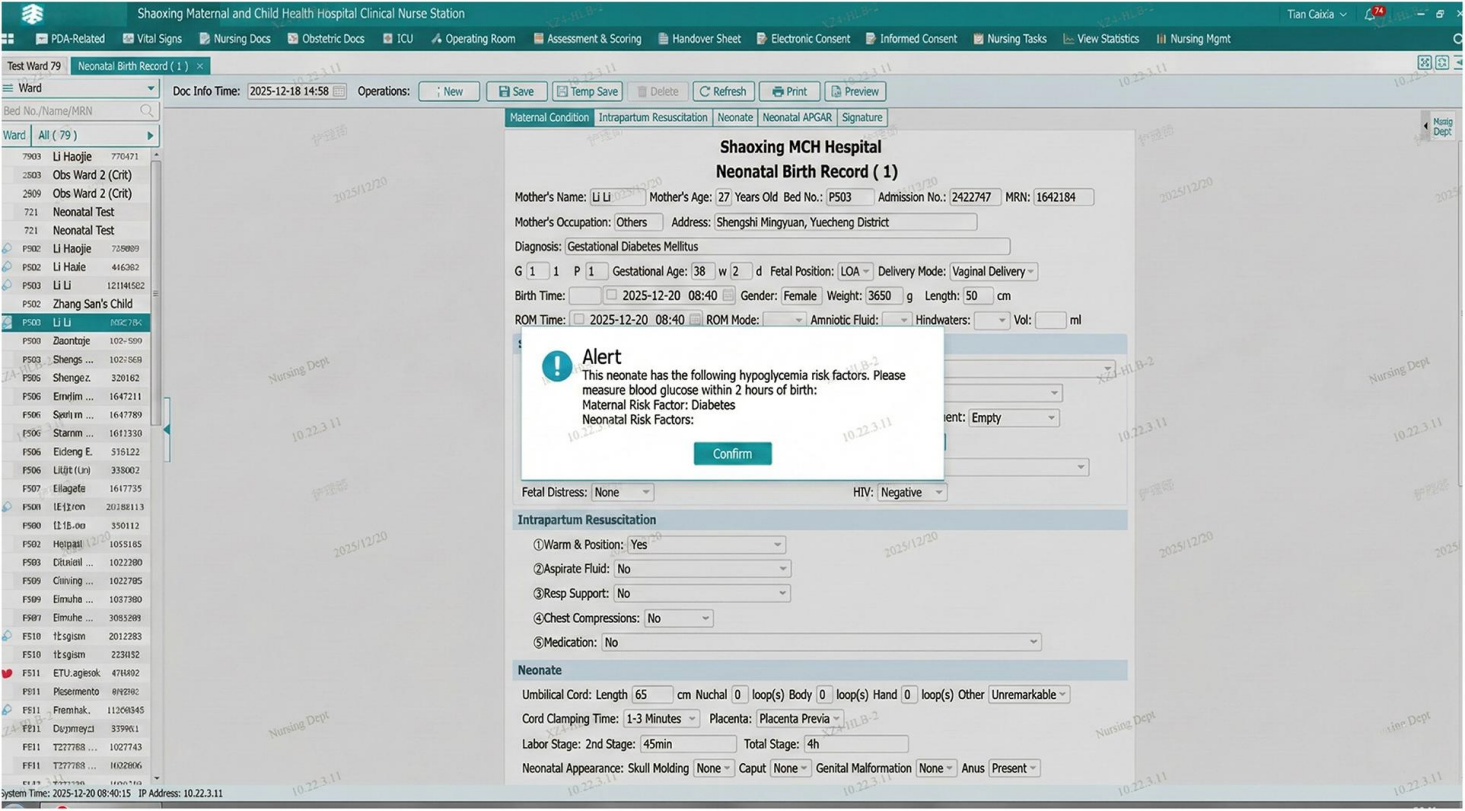
The intelligent system issuing a real-time hypoglycemia risk alert. Upon delivery, the system automatically detects maternal diabetes from electronic records and generates a prominent warning on the birth record, specifying the risk factor and mandating a blood glucose measurement promptly after birth. (All data shown in this figure are dummy data fabricated for demonstration purposes and do not represent real patient information).

**Figure 2:**
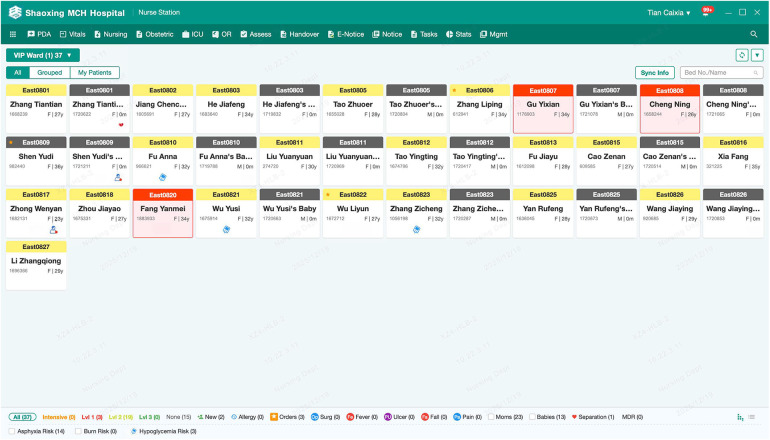
The nursing dashboard interface for centralized monitoring of mother-infant dyads at risk. The system automatically flags neonates at high risk for hypoglycemia with a distinct visual indicator (yellow highlight and a blue glucometer icon, as shown in the lower section of the interface). (All data shown in this figure, including patient bed numbers and visual indicators, are dummy data fabricated for demonstration purposes and do not represent real patient information).

**Figure 3:**
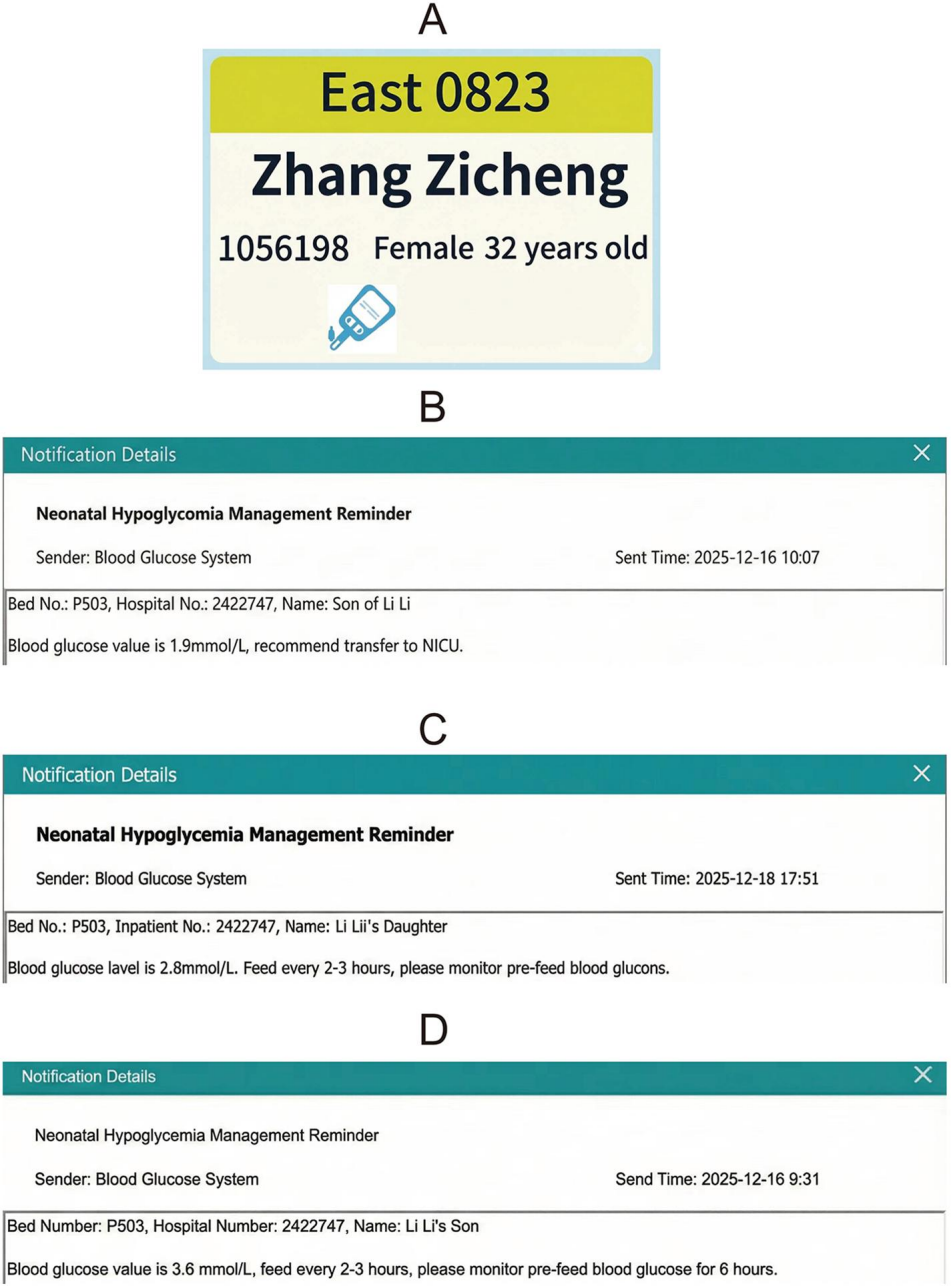
The decision-support function of the system for stratified management following blood glucose measurement. The system generates specific, tiered clinical guidance based on real-time blood glucose values: **(A)** The patient list interface where a flagged high-risk neonate (blue glucometer icon) is selected for management. **(B)** Critical Alert (Glucose 1.9 mmol/L): For values below the critical threshold (<2.2 mmol/L), the system immediately recommends transfer to the Neonatal Intensive Care Unit (NICU). **(C)** Enhanced Monitoring (Glucose 3.0 mmol/L): For intermediate values, it prescribes a feeding schedule (every 2–3 h) coupled with pre-feed glucose monitoring. **(D)** Routine Follow-up (Glucose 3.6 mmol/L): For normal values, it advises routine feeding and a single follow-up glucose check within 6 h. (All patient names, bed numbers, hospital numbers, blood glucose values, and clinical recommendations shown in this figure are dummy data fabricated for demonstration purposes and do not represent real patient information).

Figure 1 demonstrates the real-time operational state of the intelligent decision support system at the clinical interface. By interfacing directly with the hospital's electronic medical records, the system automatically identifies the presence of the high-risk factor gestational diabetes mellitus in the mother and immediately triggers a structured alert module within the neonatal birth record interface. The alert states, “This neonate has high-risk factors for hypoglycemia. Please measure blood glucose within 2 h after birth,” and lists the identified risk category as ‘infant of a diabetic mother’. This process illustrates three core capabilities of the system: 1) data integration and automated judgment, eliminating the need for manual chart review by nursing staff; 2) precise, real-time early warning, delivering standardized monitoring guidance at the earliest point after birth; and 3) integration into the clinical workflow**,** merging risk management prompts with the routine nursing documentation system to ensure the standardized initiation and timely execution of the high-risk newborn screening protocol. Figure 2 displays the nursing interface for an entire mother-infant rooming-in ward. Neonates identified as high-risk for hypoglycemia are visually flagged with a blue glucometer icon on a yellow background (see Figure 3A), indicating the need for prompt blood glucose monitoring**.** This provides nursing staff with an immediate, at-a-glance overview of the ward, enabling prioritization of monitoring tasks. The interface also aggregates and displays the total number of neonates identified as at risk for hypoglycemia. Figure 3 illustrates the stratified decision-support function of the intelligent management system following blood glucose measurement, showing different clinical management recommendations triggered by specific blood glucose value intervals. It presents the system's workflow of automated judgment and delivery of structured guidance based on evidence-based rules. Figure 3B (glucose 1.9 mmol/L) demonstrates the automatic generation of the highest-level alert when a monitored blood glucose value falls below the critical threshold (<2.2 mmol/L). The interface displays “Blood glucose is 1.9 mmol/L. Recommend transfer to the Neonatal Intensive Care Unit (NICU).” This prompt aligns with clinical guideline requirements for urgent intervention in severe hypoglycemia, reflecting the system's role in timely critical value management. Figure 3C (glucose 3.0 mmol/L) shows the management strategy for blood glucose values within the intermediate range (2.6–3.3 mmol/L). The system prompts, “Blood glucose is 3.0 mmol/L. Feed every 2–3 h; please monitor pre-feed blood glucose every 3 h.” This recommendation follows the monitoring principles for neonates with mildly low or low-normal glucose values, balancing risk surveillance with routine care by ensuring feeding support alongside a scheduled recheck. Figure 3D (glucose 3.6 mmol/L) illustrates the management protocol for blood glucose values within the normal range (≥3.3 mmol/L). The system prompts, “Blood glucose is 3.6 mmol/L. Feed every 2–3 h; please monitor pre-feed blood glucose within 6 h.” For neonates with an initially normal blood glucose level, the system continues routine feeding advice while extending the interval for the next monitoring, reflecting a risk-based, differentiated monitoring frequency designed to reduce unnecessary testing.

### Results of decision support system effectiveness validation

Two comparative cohorts were included in this study. In the pre-post self-controlled analysis, the historical baseline control group (January–March 2024, prior to CDSS implementation) included 522 high-risk neonates, while the CDSS-implemented group (April–June 2024, same wards after CDSS implementation) included 417 high-risk neonates. No notable differences were observed between the two groups in baseline variables, including maternal age (30.52 ± 4.49 vs. 30.15 ± 4.67, *P* = 0.217), infant sex (*P* = 0.888), gestational hypertension (*P* = 0.096), thyroid disorders (*P* = 0.200), gestational diabetes mellitus (*P* = 0.610), obesity (*P* = 0.130), pre-delivery medications (*P* = 0.152), intrauterine growth restriction (*P* = 0.772), intrapartum hypoxia (*P* = 0.569), large for gestational age (*P* = 0.102), preterm birth (*P* = 0.080), macrosomia (*P* = 0.308), and low birth weight (*P* = 1.000), indicating good comparability between the two groups. A difference was observed for small for gestational age (7.28% vs. 3.60%, *P* = 0.015). In the concurrent parallel control analysis, the non-CDSS group (wards with delayed implementation) included 389 neonates, while the CDSS group (wards with timely implementation) included 352 neonates. No notable differences were found between the two groups in any baseline variables (all *P* > 0.05), including maternal age (*P* = 0.444), infant sex (*P* = 0.099), gestational hypertension (*P* = 0.823), thyroid disorders (*P* = 0.554), gestational diabetes mellitus (*P* = 0.187), obesity (*P* = 0.366), pre-delivery medications (*P* = 1.000), intrauterine growth restriction (*P* = 0.083), intrapartum hypoxia (*P* = 0.855), small for gestational age (*P* = 0.150), large for gestational age (*P* = 0.270), preterm birth (*P* = 0.557), macrosomia (*P* = 0.880), and low birth weight (*P* = 0.116), suggesting that the two groups were well-balanced at baseline. In the pre-post self-controlled analysis (Table 1), the incidence of neonatal hypoglycemia in the CDSS-implemented group was 5.76% (24/417), which was lower than that in the non-implemented group (11.88%, 62/522) (*χ*^2^ = 10.44, *P* < 0.05). In the concurrent parallel control analysis (Table 2), the incidence of neonatal hypoglycemia in the CDSS group was 5.40% (19/352), also lower than that in the non-CDSS group (9.25%, 36/389) (*χ*^2^ = 4.00, *P* < 0.05). In summary, both the pre-post self-controlled analysis and the concurrent parallel control analysis demonstrated that the implementation of the CDSS was associated with a reduction in the incidence of neonatal hypoglycemia among high-risk neonates in mother-infant rooming-in settings.

**Table 1:** Pre-post self-controlled analysis of CDSS implementation.

Variables	Pre-CDSS (*n* = 522)	Post-CDSS (*n* = 417)	Statistic	*P*
Maternal age, Mean ± SD	30.52 ± 4.49	30.15 ± 4.67	*t* = 1.24	0.217
Infant sex, *n* (%)			*χ*^2^ = 0.02	0.888
Male infant	283 (54.21)	228 (54.68)		
Female infant	239 (45.79)	189 (45.32)		
Gestational hypertension, *n* (%)			χ^2^ = 2.77	0.096
No	448 (85.82)	373 (89.45)		
Yes	74 (14.18)	44 (10.55)		
Thyroid disorders in pregnancy, *n* (%)			χ^2^ = 1.64	0.200
No	443 (84.87)	366 (87.77)		
Yes	79 (15.13)	51 (12.23)		
Gestational diabetes mellitus, *n* (%)			χ^2^ = 0.26	0.610
No	350 (67.05)	273 (65.47)		
Yes	172 (32.95)	144 (34.53)		
Obesity in pregnancy, *n* (%)			χ^2^ = 2.29	0.130
No	485 (92.91)	376 (90.17)		
Yes	37 (7.09)	41 (9.83)		
Pre-delivery medications within 24 h, *n* (%)			χ²=2.06	0.152
No	509 (97.51)	412 (98.80)		
Yes	13 (2.49)	5 (1.20)		
Intrauterine growth restriction, *n* (%)			χ^2^ = 0.08	0.772
No	487 (93.30)	391 (93.76)		
Yes	35 (6.70)	26 (6.24)		
Intrapartum hypoxia, *n* (%)			χ^2^ = 0.32	0.569
No	436 (83.52)	354 (84.89)		
Yes	86 (16.48)	63 (15.11)		
Small for gestational age, *n* (%)			χ^2^ = 5.90	0.015
No	484 (92.72)	402 (96.40)		
Yes	38 (7.28)	15 (3.60)		
Large for gestational age, *n* (%)			χ^2^ = 2.68	0.102
No	485 (92.91)	375 (89.93)		
Yes	37 (7.09)	42 (10.07)		
Preterm birth, *n* (%)			χ^2^ = 3.06	0.080
No	517 (99.04)	407 (97.60)		
Yes	5 (0.96)	10 (2.40)		
Macrosomia, *n* (%)			χ^2^ = 1.04	0.308
No	476 (91.19)	372 (89.21)		
Yes	46 (8.81)	45 (10.79)		
Low birth weight, *n* (%)			χ^2^ = 0.00	1.000
No	517 (99.04)	413 (99.04)		
Yes	5 (0.96)	4 (0.96)		
Neonatal hypoglycemia, *n* (%)			χ^2^ = 10.44	0.001
No	460 (88.12)	393 (94.24)		
Yes	62 (11.88)	24 (5.76)		

*t*, *t*-test, χ^2^, Chi-square test; SD, standarddeviation.

**Table 2:** Concurrent parallel control analysis of CDSS implementation.

Variables	Non-CDSS (*n* = 389)	CDSS (*n* = 352)	Statistic	*P*
Maternal age, Mean ± SD	29.94 ± 4.33	30.18 ± 4.01	*t* = −0.77	0.444
Infant sex, *n* (%)			χ^2^ = 2.73	0.099
Male infant	193 (49.61)	196 (55.68)		
Female infant	196 (50.39)	156 (44.32)		
Gestational hypertension, *n* (%)			χ^2^ = 0.05	0.823
No	345 (88.69)	314 (89.20)		
Yes	44 (11.31)	38 (10.80)		
Thyroid disorders in pregnancy, *n* (%)			χ^2^ = 0.35	0.554
No	330 (84.83)	293 (83.24)		
Yes	59 (15.17)	59 (16.76)		
Gestational diabetes mellitus, *n* (%)			χ^2^ = 1.74	0.187
No	259 (66.58)	218 (61.93)		
Yes	130 (33.42)	134 (38.07)		
Obesity in pregnancy, *n* (%)			χ^2^ = 0.82	0.366
No	363 (93.32)	334 (94.89)		
Yes	26 (6.68)	18 (5.11)		
Pre-delivery medications within 24 h, *n* (%)			χ^2^ = 0.00	1.000
No	385 (98.97)	348 (98.86)		
Yes	4 (1.03)	4 (1.14)		
Intrauterine growth restriction, *n* (%)			χ^2^ = 3.01	0.083
No	358 (92.03)	335 (95.17)		
Yes	31 (7.97)	17 (4.83)		
Intrapartum hypoxia, *n* (%)			χ^2^ = 0.03	0.855
No	342 (87.92)	311 (88.35)		
Yes	47 (12.08)	41 (11.65)		
Small for gestational age, *n* (%)			χ^2^ = 2.07	0.150
No	355 (91.26)	331 (94.03)		
Yes	34 (8.74)	21 (5.97)		
Large for gestational age, *n* (%)			χ^2^ = 1.21	0.270
No	367 (94.34)	325 (92.33)		
Yes	22 (5.66)	27 (7.67)		
Preterm birth, *n* (%)			χ^2^ = 0.35	0.557
No	380 (97.69)	346 (98.30)		
Yes	9 (2.31)	6 (1.70)		
Macrosomia, *n* (%)			χ^2^ = 0.02	0.880
No	358 (92.03)	325 (92.33)		
Yes	31 (7.97)	27 (7.67)		
Low birth weight, *n* (%)			χ^2^ = 2.48	0.116
No	380 (97.69)	349 (99.15)		
Yes	9 (2.31)	3 (0.85)		
Neonatal hypoglycemia, *n* (%)			χ^2^ = 4.00	0.045
No	353 (90.75)	333 (94.60)		
Yes	36 (9.25)	19 (5.40)		

*t*, *t*-test, χ^2^, Chi-square test; SD, standarddeviation.

### Incidence of hypoglycemia across different high-risk factor combinations

**Figure 4:**
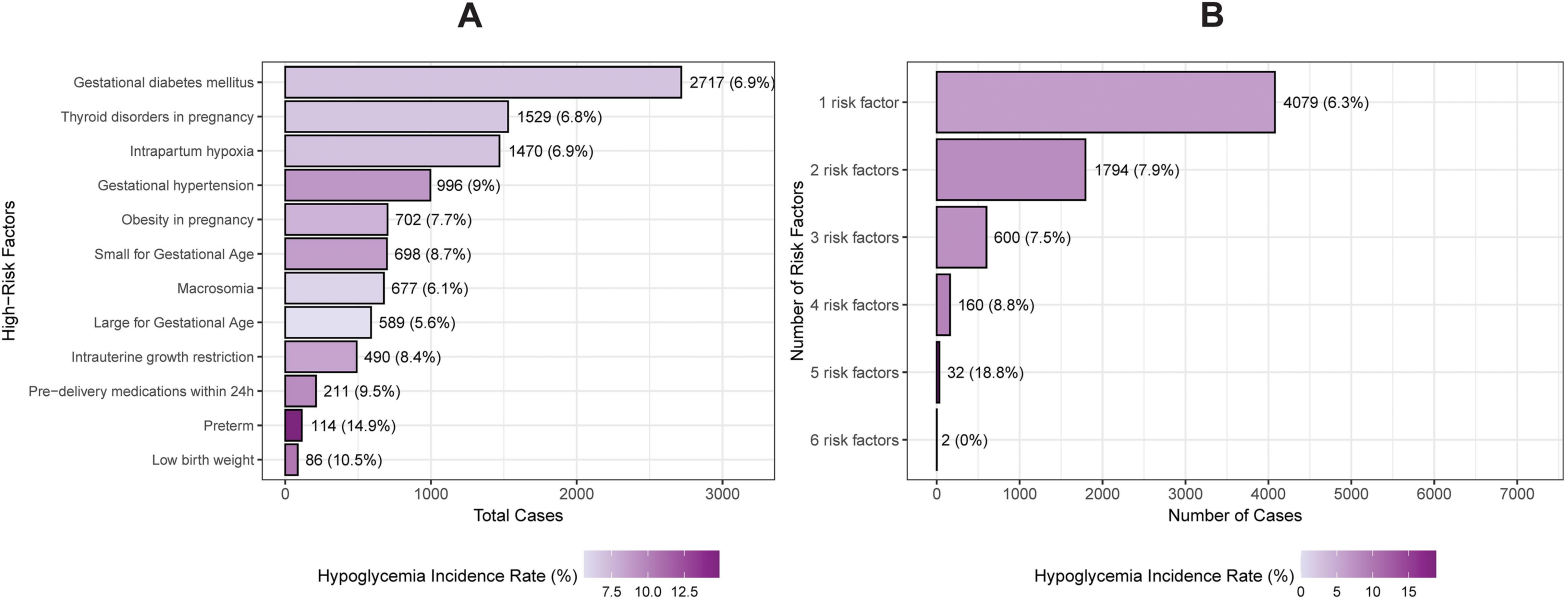
**(A)** distribution of high-risk factors and their corresponding incidence of neonatal hypoglycemia in the mother-infant rooming-in cohort under the intelligent decision support system. **(B)** The relationship between the number of high-risk factors and the incidence of neonatal hypoglycemia.

**Figure 5:**
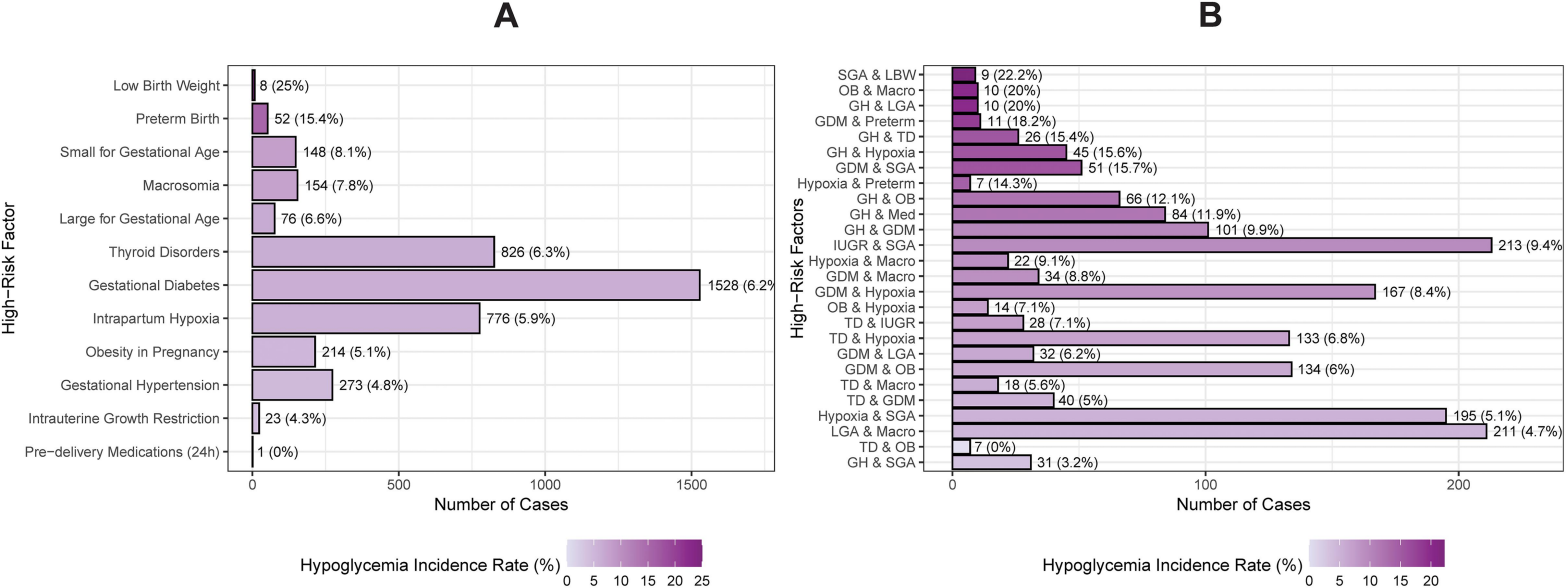
**(A)** incidence of neonatal hypoglycemia associated with individual high-risk factors among neonates with only a single risk factor. **(B)** Incidence of neonatal hypoglycemia across different two-factor high-risk combinations. GDM, gestational diabetes mellitus; GH, gestational hypertension; OB, obesity; TD, thyroid disorders; Hypoxia: intrapartum hypoxia; SGA, small for gestational age; LGA, large for gestational age; Macro, macrosomia; LBW, low birth weight; IUGR, intrauterine growth restriction; Med, pre-delivery medications within 24 h; Preterm, preterm birth.

**Figure 6:**
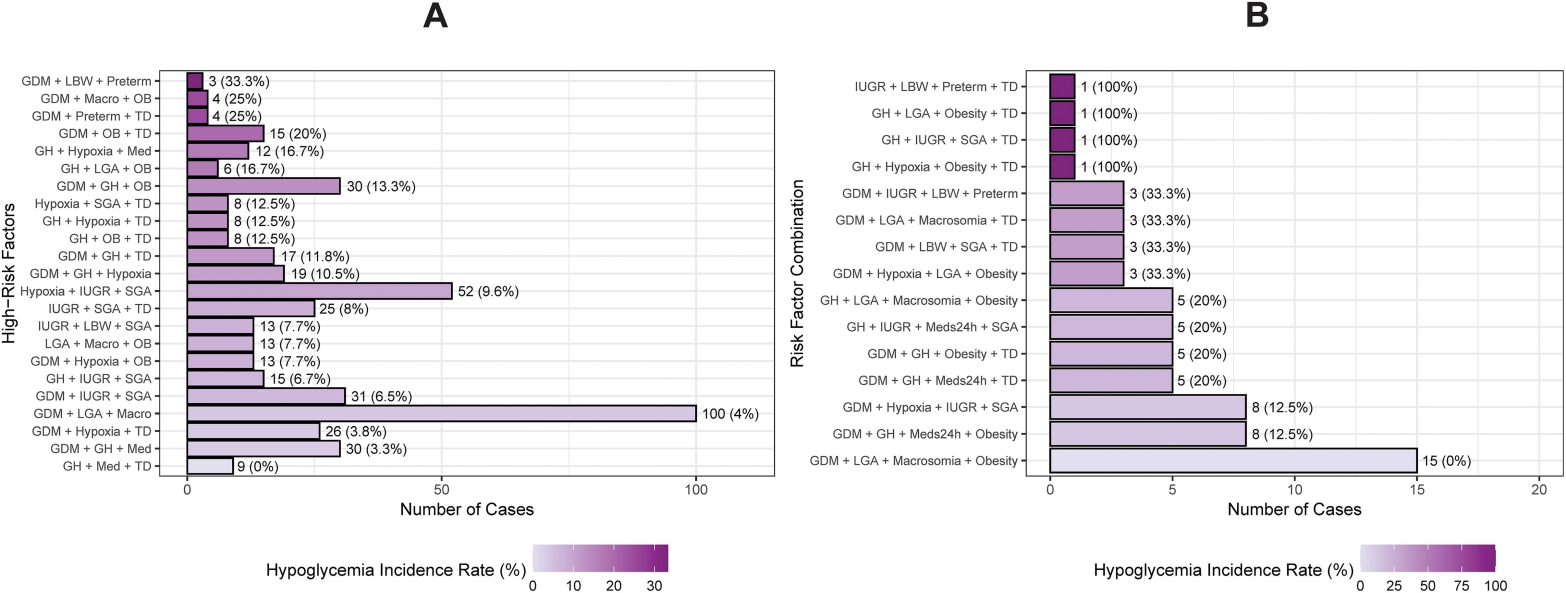
**(A)** incidence of neonatal hypoglycemia in neonates with three concurrent high-risk factors. **(B)** Analysis of hypoglycemia risk in neonates with four concurrent high-risk factors. GDM, gestational diabetes mellitus; GH, gestational hypertension; OB, obesity; TD, thyroid disorders; Hypoxia: intrapartum hypoxia; SGA, small for gestational age; LGA, large for gestational age; Macro, macrosomia; LBW, low birth weight; IUGR, intrauterine growth restriction; Med, pre-delivery medications within 24 h; Preterm, preterm birth.

Figure 4A presents the distribution of various high-risk factors and their corresponding incidence of hypoglycemia under the intelligent decision support system. This analysis reveals marked heterogeneity in both the population prevalence and risk intensity of these factors. Gestational diabetes mellitus was the most prevalent factor (2,717 cases), with a hypoglycemia incidence of 6.8%, while gestational thyroid disorders (1,529 cases) had an incidence of 6.8%. Intrapartum hypoxia (1,470 cases, 6.9%) and gestational hypertension (996 cases, 9.0%) were also common. Notably, certain less frequent factors were associated with substantially higher risks: preterm birth, though only 114 cases, had the highest incidence at 14.9%; small for gestational age (698 cases, 8.7%) and intrauterine growth restriction (490 cases, 8.4%) also showed elevated incidence. Large for gestational age (589 cases, 5.6%) and macrosomia (677 cases, 6.1%) showed relatively lower incidence. Figure 4B presents the overall trend of hypoglycemia incidence in relation to the cumulative number of high-risk factors. The majority of cases had a single risk factor (4,079 cases), with an incidence of 6.3%. As the number of concurrent risk factors increased, case counts progressively decreased: 1,794 cases with two factors (incidence 7.9%), 600 with three (7.5%), 160 with four (8.8%), 32 with five (18.8%), and 2 with six (0%). The incidence did not exhibit a simple linear increase with the number of factors; a marked increase to 18.8% was observed in the subgroup with five concurrent factors. Given the small sample sizes in higher-order combination subgroups, these findings should be interpreted with caution. Figure 5A analyzes the incidence associated with individual high-risk factors among 4,079 neonates with only a single risk factor. The analysis demonstrates substantial heterogeneity in risk levels across different factors. Low birth weight (25.0%) and preterm birth (15.4%) showed the highest incidences. The incidences for small for gestational age (8.1%) and macrosomia (7.8%) were moderate. Gestational diabetes mellitus (1,528 cases, 6.2%), gestational thyroid disorders (6.3%), and intrapartum hypoxia (5.9%) showed lower incidences. Gestational hypertension (4.8%) and intrauterine growth restriction (4.3%) presented the lowest risks. The average incidence in this single-risk-factor population was 6.3%, with the risk differential between factors reaching several-fold. Figure 5B focuses on neonates with two concurrent high-risk factors. The combination with the highest observed incidence was “small for gestational age with low birth weight” (22.2%), followed by “obesity with macrosomia” and “gestational hypertension with large for gestational age” (both 20.0%). “GDM with preterm birth” had an incidence of 18.2%, and “GDM with small for gestational age” 15.4%. Not all combinations were associated with elevated risk: “GDM with LGA” had an incidence of 6.2%, “thyroid disorders with GDM” 5.1%, and “gestational hypertension with SGA” 0%. The risk range for two-factor combinations was wide (0%–22.2%). Figure 6A analyzes neonates with three concurrent high-risk factors. The combination “GDM + low birth weight + preterm birth” (*n* = 3) had the highest observed incidence at 33.3%. Combinations “GDM + macrosomia + obesity” and “GDM + preterm birth + thyroid disorders” each had an incidence of 25.0%. Combinations such as “gestational hypertension + intrapartum hypoxia + peripartum medication” (16.7%) and “GDM + gestational hypertension + obesity” (13.3%) also showed elevated incidence. The most frequent triple combination “intrapartum hypoxia + IUGR + SGA” (*n* = 52) had an incidence of 9.6%, while “GDM + LGA + macrosomia” (*n* = 100) had an incidence of 4.0%. Given the small sample sizes in many triple-factor combinations, these results should be interpreted with caution. Figure 6B examines neonates with four concurrent high-risk factors (total 160 cases). The overall hypoglycemia incidence was 8.8%, higher than the overall incidence for high-risk neonates (6.3%). Among the 63 observed four-factor combinations, hypoglycemia events were reported in 14 combinations. Due to the limited sample size for quadruple and higher-order combinations, incidence estimates for specific combinations should be interpreted with caution.

### Analysis of trends in serial glucose monitoring and hypoglycemia dynamics

**Figure 7:**
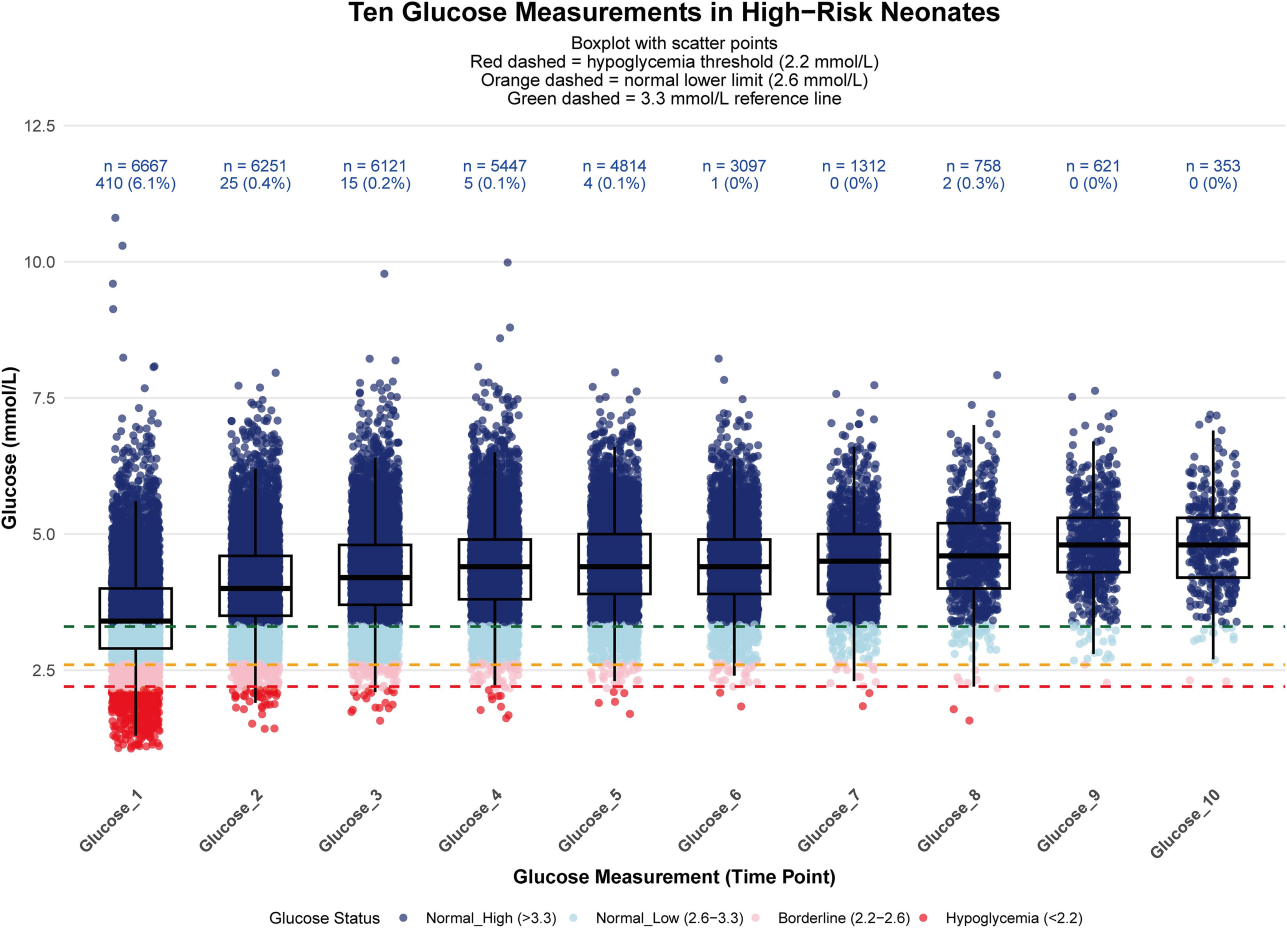
Dynamic changes in serial blood glucose measurements and the incidence of hypoglycemia among high-risk neonates under standardized management.

Figure 7, through a combination of box plots overlaid with scatter points, visually presents the dynamic trends of serial blood glucose measurements across 10 consecutive time points and the evolving pattern of hypoglycemia incidence within the high-risk neonatal cohort under the standardized management of the intelligent system. To ensure transparency in cohort construction, we report the following source population data: during the study period, there were 15,388 total births at our hospital, of which 12,890 neonates were admitted to mother-infant rooming-in wards. Among these, 6,667 neonates (51.7%) were flagged by the system as high-risk for hypoglycemia and constituted the final analyzed cohort. No cases were excluded due to incomplete data, as all core variables were derived from required fields in the electronic medical record with 100% completeness. It should be noted that neonates requiring immediate NICU transfer at birth (e.g., for severe respiratory distress or extreme prematurity) were not admitted to rooming-in wards and were therefore excluded from the cohort before any glucose monitoring occurred. This exclusion may have biased the hypoglycemia incidence estimates downward, as these excluded infants may have been at higher risk for hypoglycemia. Three reference lines in the figure denote key glycemic thresholds: a red dashed line (2.2 mmol/L, the diagnostic cutoff for hypoglycemia), an orange dashed line (2.6 mmol/L, the lower limit of normal), and a green dashed line (3.3 mmol/L, the reference standard). Overall, as the monitoring timepoints progressed (from the 1st to the 10th measurement), the median blood glucose level showed a steady increase and stabilization, while the data distribution range (represented by the box and whiskers) gradually narrowed, indicating effective overall glycemic regulation and reduced variability under systematic management intervention**.** However**,** the marked decline in hypoglycemia incidence after the first measurement must be interpreted cautiously. The incidence was highest at the first measurement (6.1%, corresponding to *n* = 410 cases), which aligns with the clinical characteristic of early postnatal glycemic instability in high-risk infants. From the second measurement onward, the incidence dropped sharply to 0.4% and remained at an extremely low level (≤0.3%) in subsequent measurements, with no hypoglycemic events observed at most timepoints from the 6th measurement onwards. Nevertheless, it is important to note that these later measurements were conditional on prior clinical pathways: infants with a first blood glucose measurement <2.2 mmol/L were transferred to the NICU and thus exited the rooming-in cohort, leading to a reduced denominator for subsequent time points. The denominators for each time point varied accordingly.

## Discussion

The data from this study indicate that under standardized management, the overall incidence of hypoglycemia in high-risk neonates was 6.3%, and the incidence demonstrated a non-linear correlation with the number of high-risk factors. Notably, neonates with five concurrent high-risk factors, representing a mere 0.48% of the total cohort, exhibited a significantly elevated hypoglycemia incidence of 18.8%, a finding consistent with the cumulative effect of risk factors reported by Wackernagel et al. (18). Granular analysis further revealed that the degree of risk is determined not only by the quantity of factors but more critically by the specific type of factor combination. For instance, preterm birth as an independent risk factor was associated with a high hypoglycemia incidence of 15.4%, which aligns closely with the underlying physiology of immature glycogen stores and hepatic gluconeogenic capacity in the fetus (19). Significantly, when “gestational diabetes mellitus” coexisted with “small for gestational age,” the hypoglycemia incidence (15.4%) was markedly higher than that for either factor alone, suggesting a pathophysiological synergy between maternal metabolic dysregulation and fetal growth restriction—a finding that deepens the discussion by Adamkin concerning the interaction of high-risk factors (20). Data from serial glucose monitoring showed that the incidence of hypoglycemia dropped sharply from 6.1% at the first measurement to below 0.4% thereafter. Although this decline partially reflects the transfer of neonates with critically low initial glucose values to the neonatal intensive care unit (NICU), the sustained low incidence among those remaining in the rooming-in cohort underscores the crucial role of continued monitoring and timely intervention, a concept aligned with recent studies emphasizing that systematic management can improve clinical outcomes (21).

Current international research primarily focuses on hypoglycemia management within the NICU, while systematic management studies specifically targeting the mother-infant rooming-in setting are relatively scarce. The intelligent management system developed in this study innovatively achieves closed-loop, full-process management from automatic risk identification and dynamic monitoring to stratified decision-making—a comprehensive management model rarely reported in the existing literature. Compared with traditional single-parameter alert systems, the core advantage of this system lies in its ability to translate complex, multi-branch clinical pathways into executable, standardized operational workflows and to enable real-time bidirectional communication via mobile nursing terminals. This design and implementation model aligns with the prevailing trend of transforming clinical practice through evidence-based, intelligent decision support tools that are seamlessly integrated with electronic health records (22). Particularly noteworthy is that while optimizing the clinical pathway, the system significantly reduces the working memory load of healthcare providers through intelligent reminders and decision support—a user-centric design of great importance for enhancing clinical adherence (23). The system's multi-modal alerts and automated escalation mechanisms are inherently directed at preventing monitoring delays, suggesting potential value for patient safety, although formal adverse event reporting was not encompassed within this study. However, the 51% high-risk flagging rate observed in our cohort deserves careful consideration. This high proportion reflects the broad inclusion criteria of the Chinese national expert consensus, which encompasses common conditions such as gestational diabetes mellitus and thyroid disorders, and may be further inflated by our hospital's role as a regional referral center. While broad screening ensures that few at-risk neonates are missed, it also means that many infants with only marginal risk are subjected to repeated blood glucose measurements. Our finding that the hypoglycemia incidence after the first measurement dropped to ≤0.4% lends support to this concern, as it suggests that a substantial proportion of high-risk-labeled neonates may undergo unnecessary heel pricks. The sharp decline in hypoglycemia incidence after the first measurement, coupled with the overall low detection rate in certain subgroups, raises the concern that these procedures cause pain and may disrupt mother-infant attachment. This underscores the importance of identifying which risk factors truly warrant serial monitoring, and our exploratory analysis of factor combinations represents a step toward such evidence-based risk stratification.

This study is a single-center, retrospective observational investigation. Although the sample size is substantial, the findings may be influenced by selection bias. The limited sample size for certain rare high-risk factor combinations affects the accuracy of statistical inferences, necessitating future multicenter collaboration to expand the cohort and validate the results (24). Secondly, the system currently employs a fixed-rule-based decision logic, which ensures standardized execution but lacks the capacity for dynamic adjustment based on individual characteristics. With advancements in medical artificial intelligence technology, future research could explore integrating machine learning algorithms into the system to develop personalized risk prediction models by analyzing real-time monitoring data (25). Thirdly, this study primarily evaluated the short-term glycemic management effectiveness of the system and lacks follow-up data on long-term outcomes such as neurodevelopment. Given the established association between neonatal hypoglycemia and long-term neurodevelopmental outcomes (2), establishing long-term follow-up cohorts will be a crucial direction for future research. Additionally, the sustainable operation of the system depends on robust hardware support and ongoing personnel training. When promoting its adoption in resource-limited healthcare settings, it is essential to fully consider the barriers and facilitators to technology adoption to ensure its accessibility and equity (26). Finally, while our findings suggest that many neonates with only marginal risk may be over-screened under the current consensus criteria, the study was not designed to formally derive an optimized screening strategy. Future prospective studies should specifically evaluate the safety and efficacy of more targeted monitoring protocols that limit serial blood sampling to the highest-risk subgroups identified in our analysis. In conclusion, the application of the intelligent decision support system has significantly enhanced the standardization and timeliness of neonatal hypoglycemia management in mother-infant rooming-in settings. The risk stratification analysis based on the large-scale, standardized data obtained through this system provides critical evidence for the precise clinical identification of high-risk neonates, ultimately driving the field of neonatal hypoglycemia management toward greater intelligence and precision.

## Conclusion

Implementation of this intelligent CDSS significantly reduced neonatal hypoglycemia incidence in mother-infant rooming-in settings, as confirmed by both pre-post and parallel control analyses. Leveraging the large-scale standardized data generated by the system, our findings further demonstrate that hypoglycemia risk depends more on the specific types and combinations of high-risk factors than on their quantity alone. This closed-loop, guideline-driven workflow enhances clinical standardization and patient safety, providing an evidence base for precise risk stratification. Future multicenter studies integrating machine learning and long-term neurodevelopmental follow-up are warranted.

## Data Availability

The original contributions presented in the study are included in the article/Supplementary Material, further inquiries can be directed to the corresponding author.
