# Supplementary figures and images for: Closed loop construction of hypoglycemia risk management for high risk neonates in mother infant rooming in settings: a retrospective study with an embedded clinical decision support system
Source: Front Pediatr. 2026 Jul 7;14:1798686. doi: 10.3389/fped.2026.1798686 (PMC13385047; doi:10.3389/fped.2026.1798686)

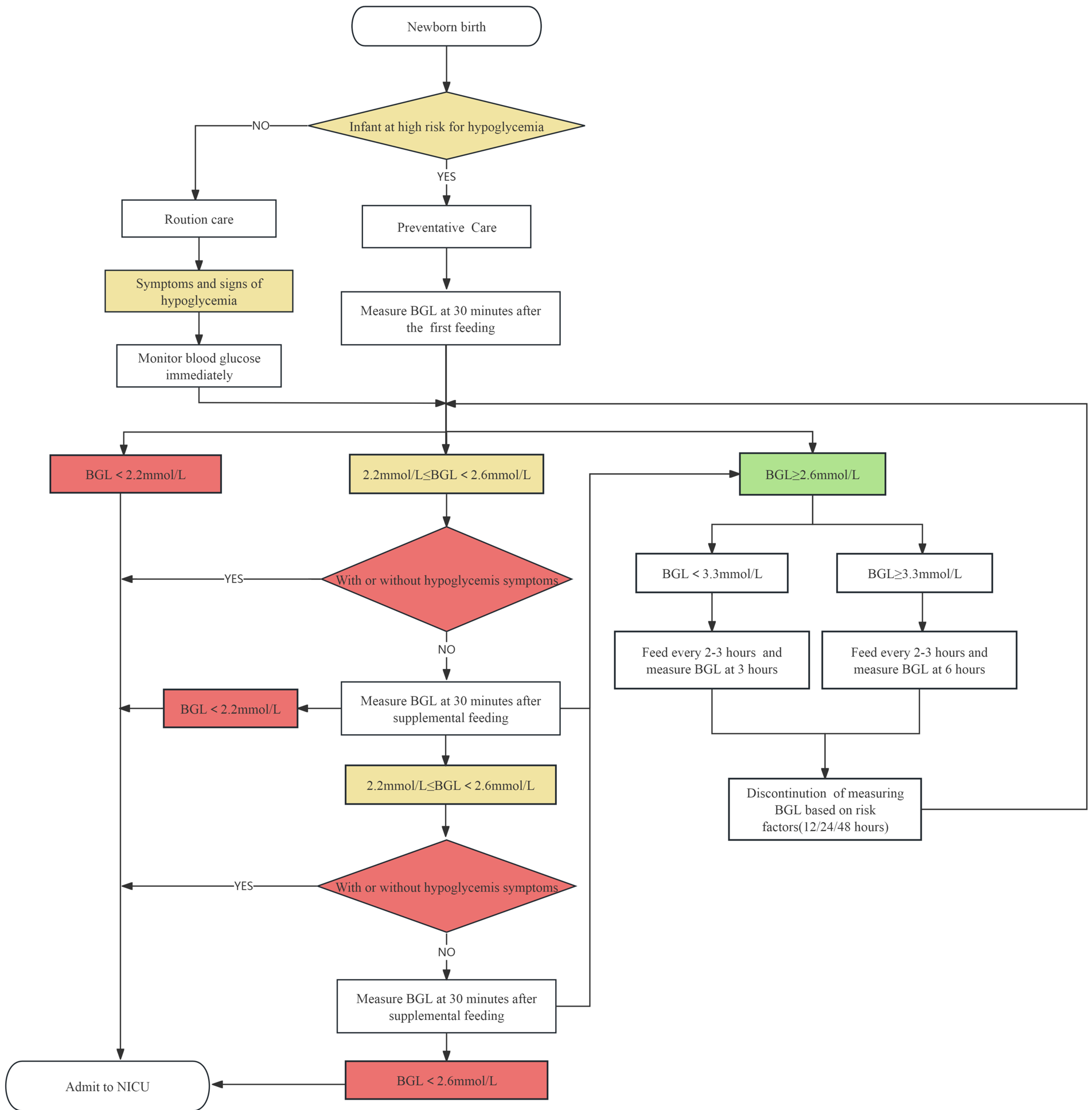

Supplement: Supplementary file 1 [file Datasheet1.pdf]
