# Supplementary material for: Closed loop construction of hypoglycemia risk management for high risk neonates in mother infant rooming in settings: a retrospective study with an embedded clinical decision support system
Source: Front Pediatr. 2026 Jul 7;14:1798686. doi: 10.3389/fped.2026.1798686 (PMC13385047; doi:10.3389/fped.2026.1798686)
Supplement: Supplementary file 2 [file Datasheet2.pdf]

Table S2. Baseline characteristics of the risk factor exploratory cohort (n = 6,667)

Variables	Non-NH (n = 6205)	NH (n = 462)	Statistic	*P*
Gestational hypertension, n(%)			χ^2=8.06	0.005
0	5299 (85.40)	372 (80.52)		
1	906 (14.60)	90 (19.48)		
Thyroid disorders in pregnancy, n(%)			χ^2=0.05	0.823
0	4780 (77.03)	358 (77.49)		
1	1425 (22.97)	104 (22.51)		
Gestational diabetes mellitus, n(%)			χ^2=0.02	0.900
0	3675 (59.23)	275 (59.52)		
1	2530 (40.77)	187 (40.48)		
Obesity in pregnancy, n(%)			χ^2=0.71	0.400
0	5557 (89.56)	408 (88.31)		
1	648 (10.44)	54 (11.69)		
Pre-delivery medications within 24h, n(%)			χ^2=2.20	0.138
0	6014 (96.92)	442 (95.67)		
1	191 (3.08)	20 (4.33)		
Intrauterine growth restriction, n(%)			χ^2=1.69	0.193
0	5756 (92.76)	421 (91.13)		
1	449 (7.24)	41 (8.87)		
Intrapartum hypoxia, n(%)			χ^2=0.00	0.988
0	4837 (77.95)	360 (77.92)		
1	1368 (22.05)	102 (22.08)		
Small for Gestational Age, n(%)			χ^2=3.96	0.047
0	5568 (89.73)	401 (86.80)		
1	637 (10.27)	61 (13.20)		
Large for Gestational Age, n(%)			χ^2=1.76	0.184
0	5649 (91.04)	429 (92.86)		
1	556 (8.96)	33 (7.14)		
Preterm, n(%)			χ^2=11.46	<.001
0	6108 (98.44)	445 (96.32)		
1	97 (1.56)	17 (3.68)		
Macrosomia, n(%)			χ^2=0.89	0.345
0	5569 (89.75)	421 (91.13)		

Variables	Non-NH (n = 6205)	NH (n = 462)	Statistic	*P*
1	636 (10.25)	41 (8.87)		
Low birth weight, n(%)			χ^2=1.69	0.194
0	6128 (98.76)	453 (98.05)		
1	77 (1.24)	9 (1.95)		

χ^2: Chi-square test, NH: Neonatal hypoglycemia.
